# Hyperglycemia induces spermatogenic disruption via major pathways of diabetes pathogenesis

**DOI:** 10.1038/s41598-019-49600-4

**Published:** 2019-09-10

**Authors:** Constanze C. Maresch, Dina C. Stute, Thomas Fleming, Jihong Lin, Hans-Peter Hammes, Thomas Linn

**Affiliations:** 1 Clinic of Urology, Pediatric Urology, and Andrology, Justus-Liebig-University, Giessen, Germany; 2 Clinical Research Unit, Centre of Internal Medicine, Justus-Liebig-University, Giessen, Germany; 3 German Center for Diabetes Research (DZD), Neuherberg, Germany; 4 V. Medical Department, Medical Faculty Mannheim, University of Heidelberg, Mannheim, Germany

**Keywords:** Diabetes complications, Male factor infertility

## Abstract

Diabetes-induced hyperglycemia has previously been shown to impact on male sub-/infertility, however, still little is known about the underlying mechanisms. In the present study we have addressed three major biochemical pathways implicated in the pathogenesis of hyperglycemia induced organ damage (the advanced glycation end product (AGE) formation pathway, the diacylglycerol-protein kinase C pathway (PKC), and the polyol pathway) in both testis and epididymis of the *Ins2*^Akita^ mouse model of Type 1 diabetes (T1DM). Hyperglycemia activated both the PKC and the polyol pathway in a significant and progressive manner within the testis, but not within the epididymis. While the AGE receptor was ubiquitiously expressed in the testis, concentrations of precursor methylglyoxal and AGE carboxymethyllysine were increased in both epididymis and testis in diabetic mice. However, AGEs did not activate intracellular pathways of ERK1, ERK2, Rela, Nrf-2, IkBkB, NFkB except CDC42, Akt1. In conclusion, two of the major pathways of hyperglycemia-induced organ damage were clearly activated within the testis of T1DM mice. This provides therapeutical opportunities in the treatment of diabetic male reproductive dysfunction.

## Introduction

Diabetes mellitus (DM) is one of the most widespread chronic diseases and a leading cause of morbidity and mortality in both developed and developing countries with an estimated prevalence of 422 million people in the year 2014^1–5^. The main characteristic of DM is hyperglycemia that results from either an autoimmune destruction of pancreatic β-cells and, thus, loss of insulin secretion (type 1 diabetes, T1DM) or insulin resistance and variable degrees of inadequate insulin secretion (type 2 diabetes, T2DM).

Three major pathways have been implicated in the pathogeneses of hyperglycemia-induced damages in various organs: polyol pathway flux, advanced glycation end product (AGE) formation, and activation of protein kinase C (PKC) isoforms through de novo synthesis of diacylglycerol (DAG)^6,7^. All of them reflect overproduction of superoxide by the mitochondrial electron transport chain, which in turn inhibits the glycolytic enzyme glyceraldehyde phosphate dehydrogenase (GAPDH), eventually causing organ damage^6–8^.

Besides others, a major complication of DM was shown to be its effect on male reproductive health, with several clinical studies reporting erectile and ejaculation dysfunction, as well as a reduction in semen volume, sperm counts, motility, and abnormal sperm morphology^9–14^. In this context, out of those three major pathways, AGE formation is known to be accelerated under diabetic conditions and was previously linked to disruptions in male fertility^13,15,16^. Therefore, various studies have reported a negative impact of AGE formation on testicular function, semen quality, and male fertility^13,16–19^.

Still little is known about the underlying mechanisms of hyperglycemia-induced male reproductive dysfunction, however, the unique characteristics of glucose metabolism within testicular cells, such as the energy independent transport though the blood-testis-barrier, make them prone to changes under diabetic conditions^9^. Thus, the ability to balance excursions of tissue glucose concentrations distinctive of diabetic patients under treatment with anti-diabetic medication plays a crucial role in the subfertility and/or infertility associated with DM.

Here we hypothesize that hyperglycemia leads to a disruption in three major glucose metabolism pathways, resulting in accumulation of the respective endproducts within the reproductive tract. Male heterozygous *Ins2*^Akita+/−^ mice, displayed a rapid and severe onset of hyperglycemia by the age of 4 weeks, which can be maintained for more than 24 weeks, thereby allowing us to directly investigate the impact of long-term hyperglycemia on the testis. We have previously shown that these mice display progressive testicular disturbance over the age of 12 to 24 weeks, affecting spermatogenesis as well as sperm density and morphology^20^. Using the same population of mice, we aimed to investigate major glucose metabolism pathways within the present study.

## Results

### Hyperglycemia activates the polyol pathway in the testis

**Figure 1:**
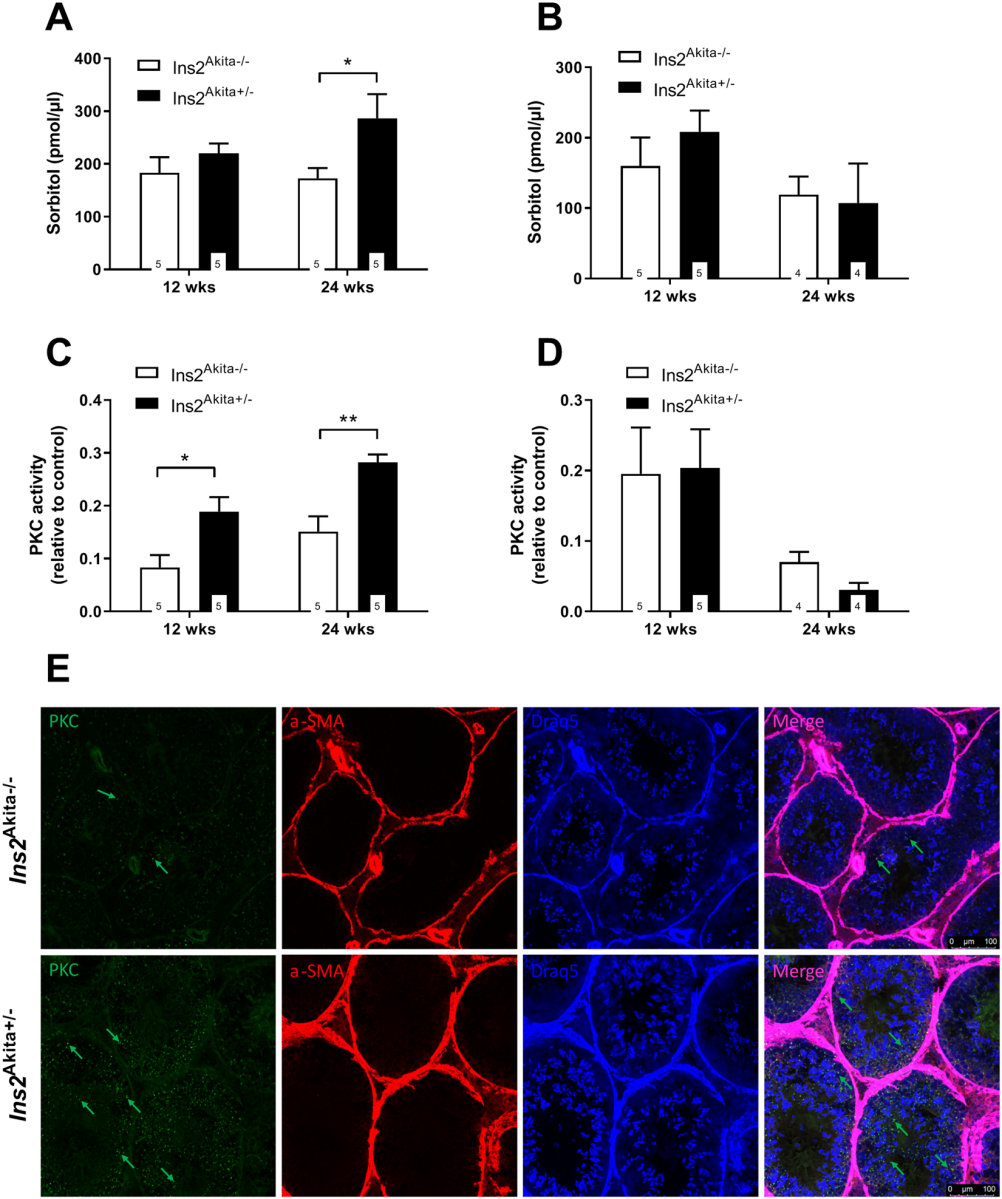
Effect of T1DM on polyol- and diacylglycerol pathway flux in *Ins2*^Akita+/−^ mice. (**A**,**B**) Polyol pathway activation was measured by sorbitol content in testis (**A**) and epididymis (**B**) using 10 µg crude protein per sample. Results from heterozygous *Ins2*^Akita^ mice were compared to non diabetic control mice at 12 and 24 weeks of age. Both testis and epididymis present with equal amounts of sorbitol, however, only within the testis hyperglycemic mice present with a significant upregulation at 24 weeks of age (172.9 ± 42.82 vs. 286.4 ± 102.0). No significant changes were observed within the epididymis. Data are expressed as mean ± SEM. Numbers in bars indicate numbers of animals per group. *p < 0.05. (**C**,**D**) Diacylglycerol pathway activation was measured by PKC activity in testis (**C**) and epididymis (**D**) using 10 µg crude protein per sample. Results from heterozygous *Ins2*^Akita^ mice were compared to non diabetic control mice at 12 and 24 weeks of age. Hyperglycemic *Ins2*^Akita+/−^ mice present with progressively increasing testicular activity of PKC rising from 0.0835 ± 0.05 vs. 0.1892 ± 0.06 (*p < 0.05) at 12 weeks of age to 0.1512 ± 0.06 vs. 0.2822 ± 0.03 (**p < 0.01) at 24 weeks of age. No significant changes were observed within the epididymis. Data are expressed as mean ± SEM. Numbers in bars indicate numbers of animals per group. (**E**) Localization of PKC in mouse testis. Testicular cryosections of *Ins2*^Akita−/−^ and *Ins2*^Akita+/−^ mice at 24 weeks of age were labeled for PKC (green), α-SMA (pink) and nuclei by Draq5 (blue). Diabetic animals show increased PKC positive signal compared to non-diabetic animals. PKC positive staining was localized in spermatocytes and round spermatids, while interstitial cells, peritubular cells and elongated and mature sperm cells were immunonegative for PKC in both groups. Scale bar is 100 µm. Images are representative for n = 4 mice examined.

To investigate the polyol pathway flux we measured sorbitol production in the testis and epididymis of *Ins2*^Akita^ mice (Fig. 1A,B). At 12 weeks of age diabetic *Ins2*^Akita+/−^ mice showed a slight increase in testicular sorbitol levels compared to non-diabetic *Ins2*^Akita−/−^ mice (183.1 ± 66.26 vs. 219.7 ± 42.45). This effect became more pronounced at 24 weeks of age, where *Ins2*^Akita+/−^ mice show a significant increase in sorbitol levels (172.9 ± 42.82 vs. 286.4 ± 102.0; p < 0.05). No changes in sorbitol content were found within the epididymis of *Ins2*^Akita+/−^ mice at any age (159.9 ± 90.37 vs. 208.6 ± 67.06 at 12 weeks of age and 119.1 ± 51.50 vs. 107.4 ± 112.0 at 24 weeks of age).

### Hyperglycemia activates the diacylglycerol pathway flux

Measurement of PKC activity was used as an indicator for the activation of the diacylglycerol pathway in testis and epididymis of *Ins2*^Akita^ mice (Fig. 1C,D). Similarly to the changes observed for the polyol pathway flux *Ins2*^Akita+/−^ mice present with progressively increasing testicular activity of PKC rising from 0.0835 ± 0.05 vs. 0.1892 ± 0.06 (p < 0.05) at 12 weeks of age to 0.1512 ± 0.06 vs. 0.2822 ± 0.03 (p < 0.01) at 24 weeks of age (Fig. 1C). No changes in PKC activity were found within the epididymis of *Ins2*^Akita+/−^ mice at any age (0.1955 ± 0.15 vs. 0.2037 ± 0.12 at 12 weeks of age and 0.07025 ± 0.03 vs. 0.03071 ± 0.02 at 24 weeks of age).

These results are further supported by immunohistochemical localization of PKC activation in mouse testis (Fig. 1E). Here, diabetic animals show increased PKC positive signal compared to non-diabetic animals at 24 weeks of age. PKC positive staining was localized in spermatocytes and round spermatids, while interstitial cells, peritubular cells and elongated and mature sperm cells were immunonegative for PKC.

### Hyperglycemia results in advanced glycation end product (AGE) formation, but does not activate AGE receptor dependent pathways

To investigate if hyperglycemia effects the production/formation of AGEs within the testis and epididymis of insulin deficient *Ins2*^Akita+/−^ mice we examined the AGE formation pathway in a comprehensive manner. Hence we first analysed the AGE precursor methylglyoxal (MG), followed by AGEs, and further their receptor RAGE as well as downstream targets.

**Figure 2:**
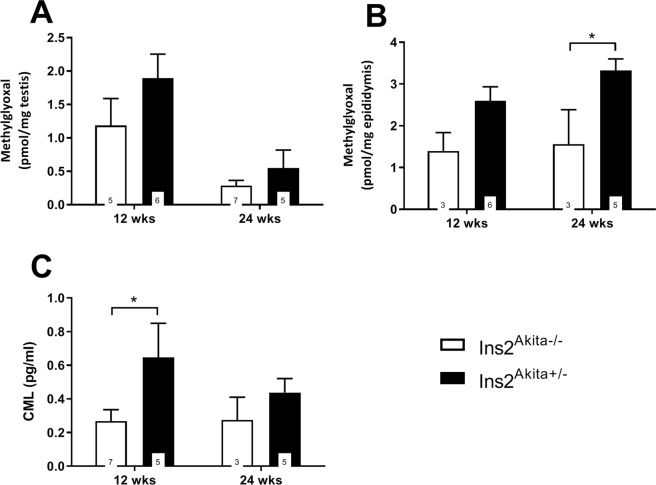
MG and CML protein in *Ins2*^Akita+/−^ mice compared to control. (**A**,**B**) Methylglyoxal content in testis (**A**) and epididymis (**B**) as determined by LC-MS/MS. *Ins2*^Akita+/−^ mice presented with elevated MG levels compared to *Ins2*^Akita−/−^ mice in both testis and epididymis at all ages, with a significant increase showing at 24 weeks in the epididymis (1.561 ± 1.43 vs. 3.323 ± 0.62, p < 0.05). Data are expressed as mean ± SEM. Numbers in bars indicate numbers of animals per group. *p < 0.05. (**C**) Protein content of CML in *Ins2*^Akita+/−^ mice compared to *Ins2*^Akita−/−^ control in testis as quantified by ELISA. Hyperglycemic *Ins2*^Akita+/−^ mice display a pronounced increase in CML at 12 weeks of age compared to non-diabetic control animals. This was not observed at 24 weeks of age. Data are expressed as mean ± SEM. Numbers in bars indicate numbers of animals per group. *p < 0.05.

MG content in testis and epididymis was analysed via MS (Fig. 2A,B). *Ins2*^Akita+/−^ mice presented with elevated MG levels compared to *Ins2*^Akita−/−^ mice in both testis and epididymis at all ages, with a significant increase showing at 24 weeks in the epididymis (1.561 ± 1.43 vs. 3.323 ± 0.62, p < 0.05).

**Figure 3:**
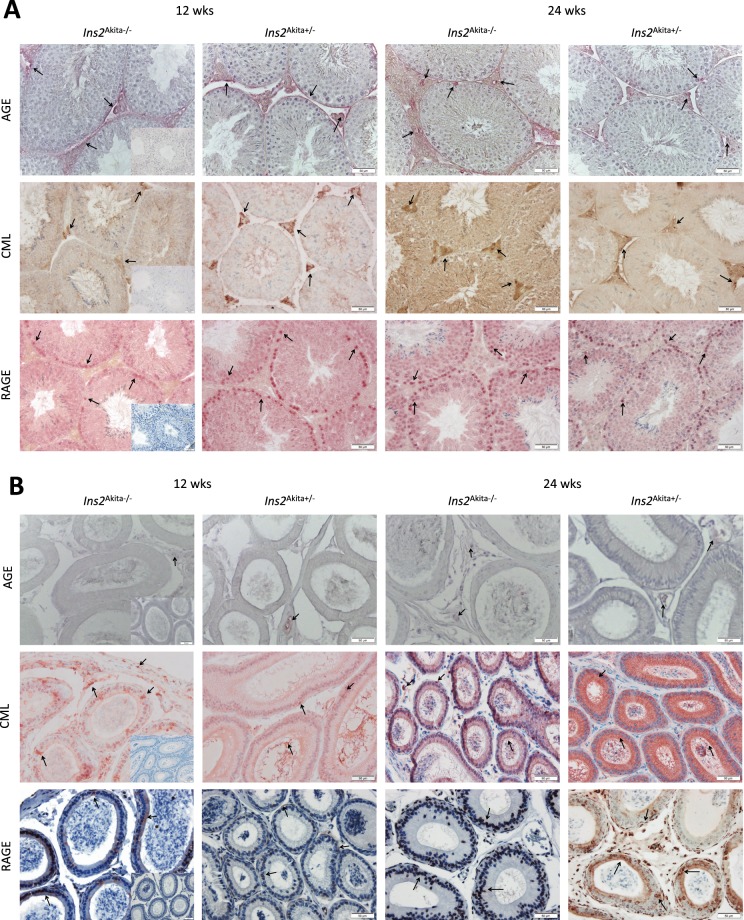
Immunohistochemical localization of AGEs, CML, and RAGE in testis (**A**) and epididymis (**B**) of diabetic *Ins2*^Akita+/−^ mice compared to non-diabetic *Ins2*^Akita−/−^ control. Within the testis AGE was expressed mainly within the interstitium in all groups at all timepoints. Moreover, there was a positive staining of the tubular basal membrane, in spermatogonia, and in Sertoli cells in some tubules. Epididymal sections showed AGE positive staining within blood vessels only. Expression of CML was limited to the testicular interstitial structures in all groups. Diabetic mice showed a strong distribution of CML along the tubular basal membrane in the testis with staining being most intense in diabetic mice at 12 weeks of age. Epididymal CML expression was low all over, but there was some staining observed in basal cells of non-diabetic mice, while *Ins2*^Akita+/−^ mice presented with pronounced staining in stereocilia and principal cells. Testicular RAGE signal was present in spermatogonia and early spermatocytes within the tubuli. Epididymal RAGE was primarily distributed in basal cells in both diabetic and control mice and in some principal cells of diabetic animals. Images are representative for n = 5 mice examined, scale bar is 50 µm. Inserted pictures on the left handside panels respresent negative control stainings.

We further analysed the distribution of AGEs within the testis and epididymis followed by protein levels of the commonly studied AGE, CML (Figs 2C and 3). Immunohistochemical analysis of AGE distribution within the testis revealed an expression mainly within the interstitium, identifying Leydig cells, macrophages, and blood vessels as AGE positive in all groups at all timepoints. Interestingly, there was also positive staining within the tubular basal membrane, in spermatogonia, and in Sertoli cells in some tubules (Fig. 3A). By contrast, epididymal sections showed AGE positive staining within blood vessels only (Fig. 3B). Similar to the AGE distribution, within the testis the commonly studied AGE CML was identified within Leydig cells, macrophages, and blood vessels (Fig. 3A). Additionally, diabetic mice showed a strong distribution of CML along the tubular basal membrane. Moreover, the staining was more pronounced in diabetic *Ins2*^Akita+/−^ mice at 12 weeks of age compared to non-diabetic mice. This observation was confirmed by CML ELISA (Fig. 2C) showing that only diabetic *Ins2*^Akita+/−^ mice display a pronounced increase in CML at 12 weeks of age compared to non-diabetic control animals. This was not observed at 24 weeks of age. Within the epididymis CML expression was observed in basal cells in *Ins2*^Akita−/−^ mice, while *Ins2*^Akita+/−^ mice presented with pronounced staining in stereocilia and principal cells.

**Figure 4:**
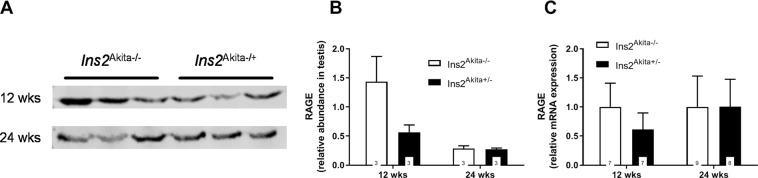
RAGE expression on protein (**A**,**B**) and mRNA level (**C**) in *Ins2*^Akita+/−^ mice compared to *Ins2*^Akita−/−^ control. (**A**) Lysates from testicular tissue were resolved in three biological replicates by SDS-PAGE and analysed by western blotting using a polyclonal antibody against RAGE. (**B**) Quantification of the data shown in (**A**). For quantitative analysis Western Blot bands were normalised to total protein loaded per lane. Stastical differences were calculated using Two-way ANOVA. No significant differences in RAGE protein expression were found between the diabetic and non-diabetic groups. Data are expressed as mean ± SEM. Numbers in bars indicate numbers of animals per group. (**C**) mRNA analysis of RAGE transcripts in mouse testis at 12 and 24 weeks of age. No significant differences in RAGE mRNA expression were found between the diabetic and non-diabetic groups. Data are expressed as mean ± SEM. Numbers in bars indicate numbers of animals per group.

In order to determine the distribution of the receptor for advanced glycation endproducts (RAGE) we stained serial sections of testicular and epididymal mouse tissue (Fig. 3). In all groups analysed testicular RAGE signal was highly present in spermatogonia and early spermatocytes cells within the tubules (Fig. 3A). Within epididymal sections RAGE was primarily distributed in basal cells in both diabetic and control animals and in principal cells in diabetic animals only (Fig. 3B). We further determined the mRNA and protein expression via qRT-PCR and Western Blot, respectively (Fig. 4). No significant differences in RAGE transcript or protein expression were found between the diabetic and non-diabetic groups.

**Figure 5:**
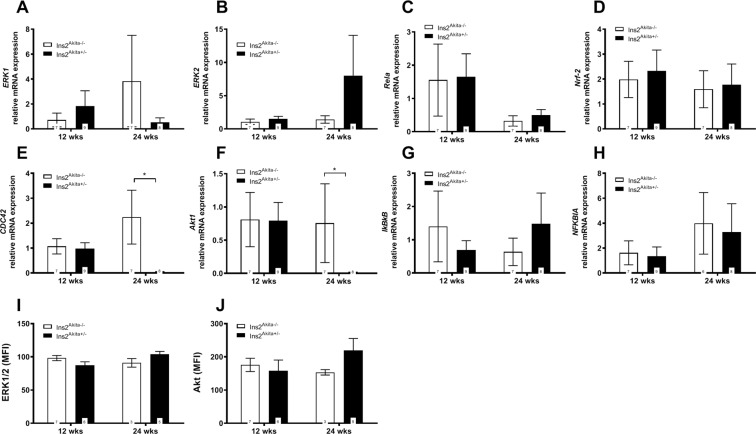
Transcript and protein levels of RAGE downstream signalling in diabetic *Ins2*^Akita+/−^ mice. (**A**–**H**) mRNA levels of ERK1 (**A**), ERK2 (**B**), Rela (**C**), Nrf-2 (**D**), CDC42 (**E**), Akt1 (**F**), IκBκB (**G**), and NFKBIA (**H**) were assessed by qRT-PCR in mouse testis of 12 and 24 weeks old *Ins2*^Akita+/−^ mice compared to controls. No significant differences were found in the expression of AGE-induced intracellular pathways, except for CDC42 and Akt1. Data are expressed as mean ± SEM. Numbers in bars indicate numbers of animals per group. (**I**,**J**) Protein levels of ERK1/2 (**I**) and Akt (**J**) were determined using the MILLIPLEX MAP TGFβ Signaling Pathway Magnetic Bead 6-Plex kit. Readouts of median fluorescent intensitiy (MFI) were quantified after normalisation to background fluorescence. While ERK1 and ERK2 were not regulated in diabetic *Ins2*^Akita+/−^ mice at any age, hyperglycemia leads to a significant upregulation in Akt at 24 weeks of age. Data expressed as median fluorescence intensity (MFI) ± SEM. Numbers in bars indicate numbers of animals per group.

**Figure 6:**
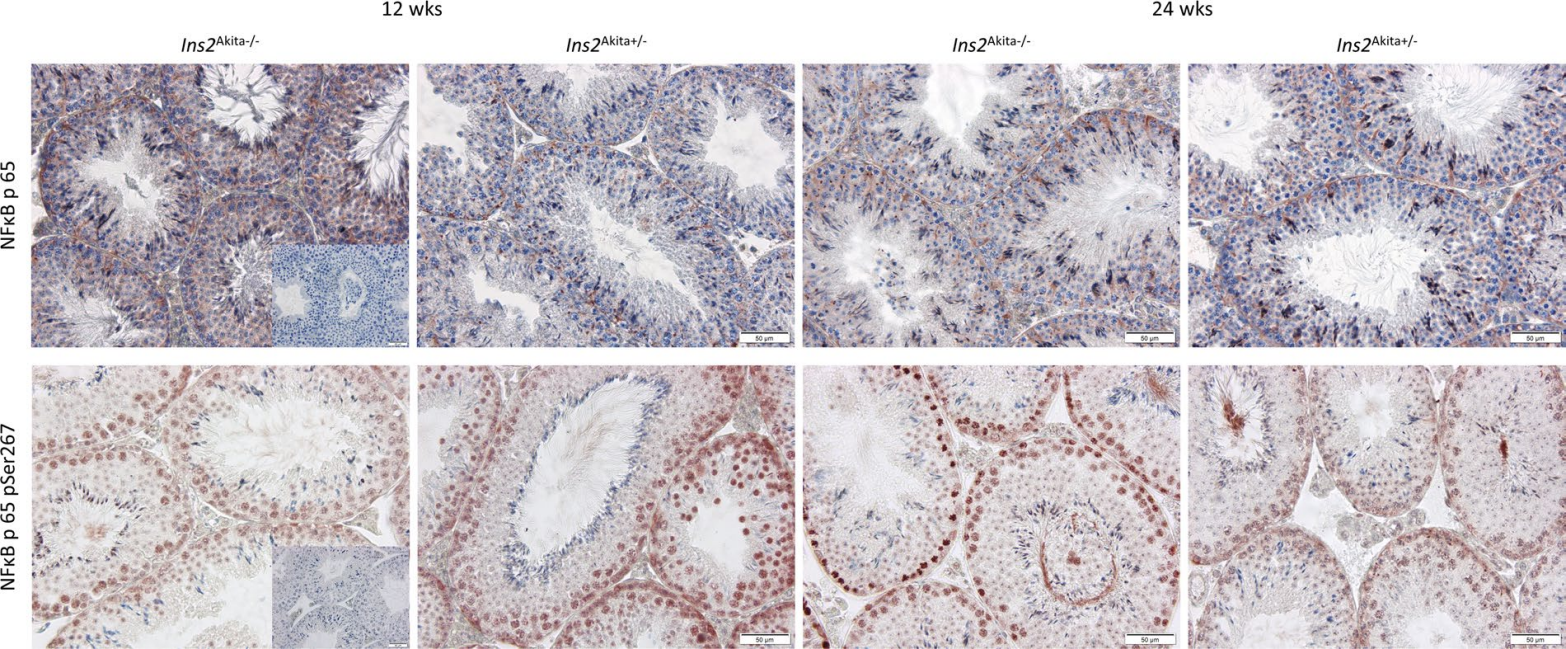
Immunohistochemical localization of NFκB p65 and phosphorylated NFκB p65 in *Ins2*^Akita+/−^ mice compared to *Ins2*^Akita−/−^ control. Histological analysis revealed a cytoplasmic distribution for NFκB p65 within Sertoli cells as well as within germ cells starting from spermatocytes to round spermatids. Here, circular, crescent-shaped vesicular structures in the cytoplasmic area adjacent to the nucleus stain positive for NFκB p65. There was no obvious difference showing between goups. Phosphorylated NFκB p65 showed a strong nuclear distribution with the staining being present in spermatogenic cells starting from spermatogonia to round spermatids and a marked staining showing in meiotic cells. Of interest, marked staining was observed in meiotic cells. Of note, it appears that at 24 weeks of age phosphorylated NFκB p65-positive cells are more distinct in the *Ins2*^Akita+/−^ mice than in the *Ins2*^Akita−/−^ mice. Images are representative for n = 5 mice examined, scale bar is 50 µm. Inserted picture on the left handside respresents negative control staining.

Next, we assessed several RAGE downstream target genes via qRT-PCR (Fig. 5). ERK1 and ERK2 were not regulated in diabetic *Ins2*^Akita+/−^ mice at all ages. This finding is in accordance with the protein analysis for the ERK1/2 complex (Fig. 5I). No significant differences were found in the expression of *Rela* and *Nrf-2*, however, genes for *CDC42* and *Akt1* were significantly downregulated in diabetic *Ins2*^Akita+/−^ mice when compared to non-diabetic control animals at 24 weeks of age. In contrast to the gene expression, Akt1 protein was significantly upregulated in diabetic *Ins2*^Akita+/−^ mice at 24 weeks of age (Fig. 5J). To examine the RAGE downstream target NFκB, we investigated both gene transcripts and protein levels. qRT-PCR for IκBκB and Nfκbia showed no regulation in IκBκB or Nfκbia for diabetic *Ins2*^Akita+/−^ mice at any age. Histological analysis revealed a cytoplasmic distribution for NFκB p65 within Sertoli cells as well as within germ cells starting from spermatocytes to round spermatids. Here, circular, crescent-shaped vesicular structures in the cytoplasmic area adjacent to the nucleus stain positive for NFκB p65. There was no obvious difference showing between goups (Fig. 6). As expected, phosphorylated NFκB p65 showed a strong nuclear distribution with the staining being present in spermatogenic cells starting from spermatogonia to round spermatids. Of interest, marked staining was observed in meiotic cells. Of note, it appears that at 24 weeks of age phosphorylated NFκB p65-positive cells are more distinct in the *Ins2*^Akita+/−^ mice than in the *Ins2*^Akita−/−^ mice (Fig. 6).

### Hyperglycemia affects cytoskeleton formation in mouse testis

**Figure 7:**
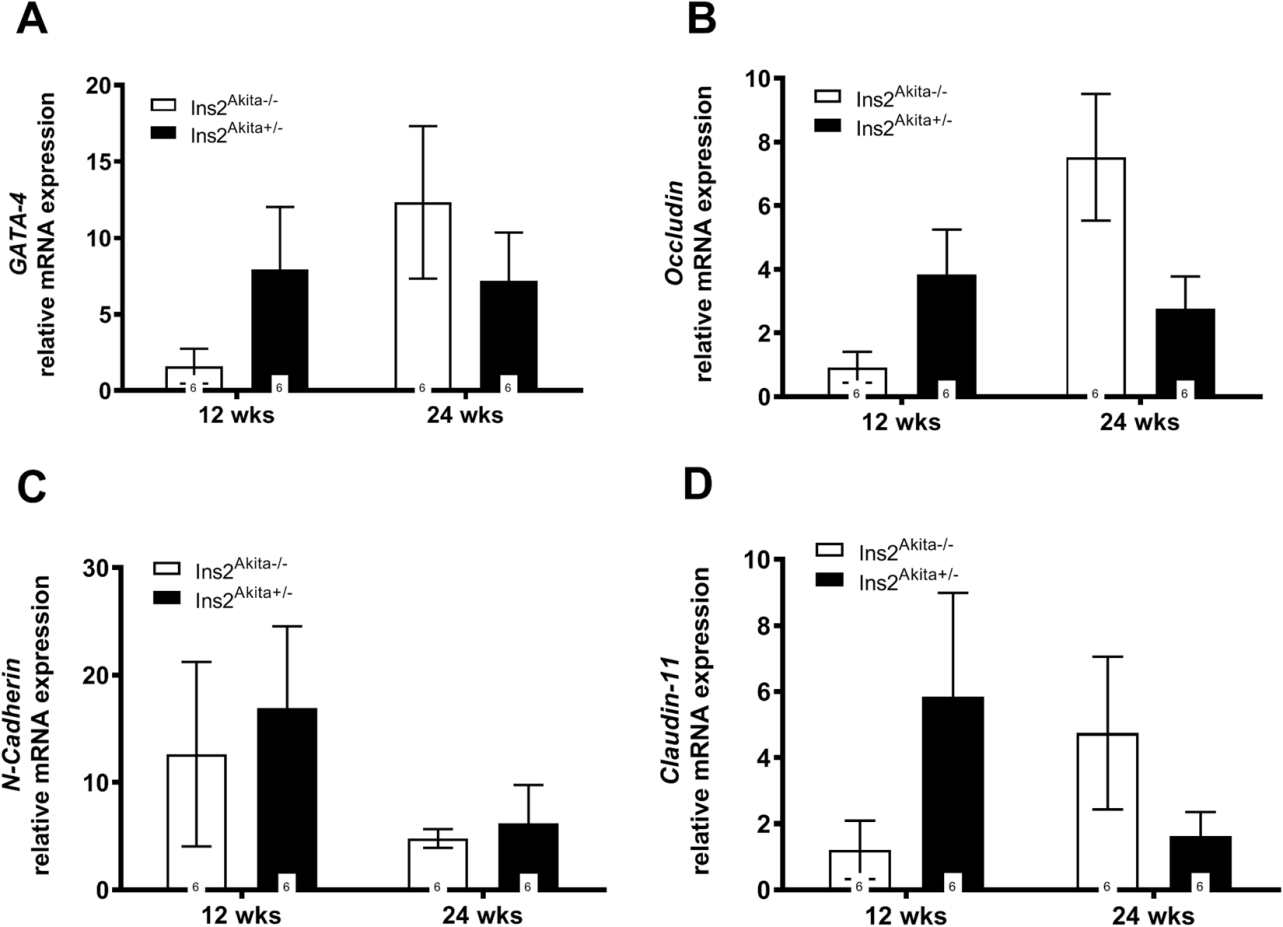
Blood-testis-Barrier related genes in diabetic *Ins2*^Akita+/−^ mice. (**A**–**D**) qRT-PCR assessment of *GATA-4* (**A**), *Occludin* (**B**), *N-Cadherin* (**C**), and *Claudin-11* (**D**) mRNA expression in mouse testis. Blood-testis-Barrier related genes did not show any significant changes, but transcripts of all genes tended to be enhanced at 12 weeks and downregulated at 24 weeks with the exception of N-cadherin which was upregulated at 24 weeks. Data expressed as mean ± SEM. Numbers in bars indicate numbers of animals per group.

The finding that Akt1 and CDC42 were regulated in diabetic mice at 24 weeks of age, led to the hypothesis that in these mice the formation of cytoskeleton components known to form the blood testis barrier might be involved. The expression of genes encoding tight junction proteins claudin 11, N-Cadherin, Gata4, and occludin was quantified by qRT-PCR, but did not show any significant changes (Fig. 7). Interestingly, transcripts of all genes tended to be enhanced at 12 weeks and downregulated at 24 weeks with the exception of N-cadherin which was upregulated at 24 weeks.

### Hyperglycemia does not affect the formation of DNA double strand breaks in mouse testis

**Figure 8:**
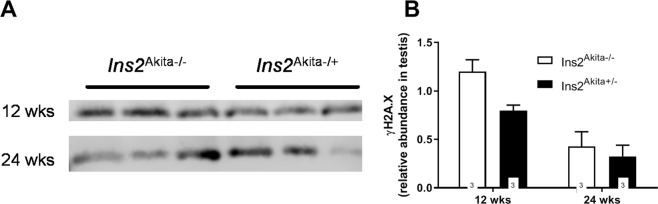
Analysis of DNA double strand breaks in the testis of diabetic *Ins2*^Akita+/−^ mice. (**A**) Lysates from testicular tissue were resolved in three biological replicates by SDS-PAGE and analysed by western blotting using a polyclonal antibody against γH2A.X. (**B**) Quantification of the data shown in (**A**). For quantitative analysis Western Blot bands were normalised to total protein loaded per lane. Stastical differences were calculated using Two-way ANOVA. No significant differences in γH2A.X protein expression were found between the diabetic and non-diabetic groups. Data are expressed as mean ± SEM. Data are representative for n = 3 mice examined.

Previously it was reported that loss of RAGE causatively linked to perpetual DNA double strand break signalling^21^. On the basis of the observed RAGE downregulation found in the present study we additionally investigated H2A.X expression which was used to reflect the presence of strand breaks. Interestingly, Ins2^Akita+/−^ mice did not present with increased DNA damage at any age (Fig. 8). Of note, no sign of DNA damage could be detected within epididymal tissue.

## Discussion

Previously, attention was raised to the correlation between hyperglycemia *per se* and male reproductive dysfunction, showing that poorly controlled blood glucose in conjunction with late stage diabetic complications reduces both sperm counts and testosterone blood levels^22^. Suggested underlying mechanisms in this context were impaired function of the hypothalamic-pituitary-gonadal axis, increased DNA damage, perturbations in the AGE/RAGE system, oxidative stress, increased endoplasmic reticulum stress, modulation of cellular pathways, impaired mitochondrial function and disrupted sympathetic innervation. However, confirmative results identifying the pathological details are still scarce.

In the present study, we examined the hypothesis that hyperglycemia resulted in a disruption of three major glucose metabolism pathways, leading in turn to an accumulation of the respective endproducts within the reproductive tract. Having demonstrated that prolonged exposure to hyperglycemia was in fact associated with progressive testicular disruption in the *Ins2*^Akita+/−^ mouse model^23^, we now present experimental evidence for the activation of two out of three major glucose metabolism pathways.

Sorbitol presented with similar levels in testis and epididymis in nondiabetic mice and a significant upregulation under hyperglycemic conditions at 24 weeks of age with concentrations rising 1.7-fold (172.9 ± 42.8 to 286.4 ± 102.0 pmol/µl) within the testis. It was previously suggested that the polyol pathway plays a significant role in testicular metabolism and spermatogenesis^24^. Thus, the enzymes of the polyol pathway, aldosereductase and sorbitol dehydrogenase were found most abundantly expressed in Sertoli and germ cells, respectively, in rodent testes^24,25^. Furthermore, as sperm can utilize fructose as sole energy product, the polyol pathway was supposed to be active in testis^26,27^. Despite this correlation, studies investigating the effects of diabetes or hyperglycemia *per se* on polyol metabolism in testis are rare. Hoshi *et al*. have investigated enzyme levels and activity of SDH in the testis of diabetic rats with no changes observed. The authors argue that this might be due to relatively high amount of fructose and an accelerated glycation reaction in this tissue^26^. Our study is the first to provide data on sorbitol content in mouse testis providing evidence that the polyol pathway is further activated in this organ under hyperglycemic conditions.

Changes in the PKC regulation were previously reported to play a crucial role in hyperglycemia-induced damages of the retina, kidney and cardiovascular system by facilitating microvascular permeability, angiogenesis, endothelial cell turnover, and leukocyte adhesion^28–30^. In this context, inhibition of the activation of the diacylglycerol pathway was shown to prevent diabetic complications^31^. Within the male reproductive system, PKC was shown to be present in testis and epididymis in various isoforms^32^. Further, the presence of PKC within the sperm head of various species indicated involvement in the acrosome reaction, supposedly via opening of calcium channels^33,34^. To the best of our knowledge there are no studies investigating the regulation of PKC within the testis under hyperglycemic conditions. Here we showed that PKC activity is significantly enhanced in testis of diabetic mice at 12 and 24 weeks of age. This effect was not observed within the epididymis. The observation provided further evidence that PKC plays a further role in sperm development and is likely to contribute to hyperglycemia-induced fertility disorders in the male.

The accumulation of AGEs is known to contribute to the development of diabetic complications such as nephropathy, retinopathy or neuropathy. Studies investigating this pathway in the context of male sub- or infertility have described that AGEs are increasingly detected in the semen of diabetic men, leading to functional sperm modifications through the production of reactive oxygen species^17^. Thus, T1DM men present with a significant increase in AGEs and lipid peroxidation in seminal plasma and spermatozoa as well as a reduced antioxidant capacity^12,17,35^. Moreover, localized in testis, epididymis and spermatozoa, RAGE was found to be significantly elevated in T1DM and directly correlated with increased DNA fragmentation in spermatozoa^16,18,35^. However, functional studies investigating the underlying mechanisms of glycation induced damage within the male reproductive tract are still missing. Here we show that diabetic *Ins2*^Akita^ mice present with elevated levels of the AGE precursor MG both in the testis and epididymis as well as the AGE CML within the testis. Concomitantly, RAGE protein expression was decreased at 12 weeks of age while only few downstream target genes were affected at 24 weeks of age in diabetic mice. Overall, there are few studies that have assessed a quantitative RAGE distribution within the reproductive tract. While O’Neil *et al*. could not detect any changes in the RAGE distribution in spermatozoa between non-diabetic and diabetic mice^35^, both Karimi *et al*. and Mallidis *et al*. reported that men with diabetes mellitus express significantly higher levels of RAGE in spermatozoa. In addition, this observation was positively correlated with increased DNA fragmentation in spermatozoa^16,18^. In contrast, Miura *et al*. described a negative correlation between AGEs and RAGE in human monocytes. The expression of RAGE was significantly reduced in humans with retinopathy or nephropathy^36^. These observations show, that RAGE acts in a tissue and pathology related manner. In the present study, we were not able to confirm a differential expression of RAGE or a relation to increased DNA fragmentation and the activation of the canonical downstream target NFκB. This might partially be due to the significant downregulation of the upstream factors CDC42 and Akt1 on the transcript level. However, both of these factors are not only upstream factors for the activation of NFκB, but also necessary for the physiological course of spermatogenesis and are involved in functions such as cell growth, proliferation and apoptosis^37–41^. Thus, the decrease of the AGE receptor in the reproductive tract of *Ins2*^Akitas+/−^ and whether it supposedly directly contributes to cell damage and the observed morphological changes^23^, requires further investigatons.

A limitation of the present study is the fact that we have not shown a direct causal link between the activated glucose metabolism pathways and the testicular damage. Future studies would benefit from an intervention model for instance using the *Ins2*^Akita+/−^ mouse on a controlled insulin regime, to prove the interrelation between the activation of the polyol and PKC pathway and the observed fertility disorders. Thus, our results provide the basis of a hypothesis that could be tested in future studies to unravel hyperglycemia- induced subfertility in heterozygous *Ins2*^Akita^ mice.

## Conclusion

The present study shows that hyperglycemia induces two out of three major glucose metabolism pathways within the testis of T1DM mice. Thus, hyperglycemia activated both the PKC and the polyol pathway in a significant and progressive manner within the testis, but not within the epididymis. While the AGE receptor was ubiquitiously expressed in the testis, concentrations of precursor methylglyoxal and AGE carboxymethyllysine were increased in both epididymis and testis in diabetic mice. However, AGEs did not activate intracellular pathways of ERK1, ERK2, Rela, Nrf-2, IκBκB, NFκB except CDC42, Akt1. In summary, the data reported here indicate that addressing pathways of hyperglycemic damage in the treatment of diabetic patients may prevent or delay the development of hyperglycemia-induced male reproductive dysfunction.

## Research Design and Methods

### Animals and reagents

All animals were maintained following approval from the Animal Ethics Committee, in accordance with German Animal Welfare Act for the care and use of laboratory animals and the rules of the regulatory authorities in Baden-Württemberg (Reg. präsidium Karlsruhe, Germany). Diabetic heterozygous *Ins2*^Akita+/−^ (*Ins2*^Akita^) mice, purchased from Jackson Laboratory (Charles River Laboratories, Germany), were bred at the animal facility of the University Hospital Mannheim, Heidelberg University. Age-matched non-diabetic homozygous *Ins2*^Akita−/−^ littermates served as control. Testis and epididymis of 12- and 24-week-old *Ins2*^Akita^ mice were used. Blood glucose was monitored consecutively over the study period through punctuation of the tail using a syringe cannula via a BG Star blood glucose meter (Sanofi-Aventis Deutschland GmbH, Germany). At 8 weeks of age, approximately 50% of these mice had permanent blood glucose levels above 14.0 mmol/l and were classified as severely hyperglycemic, insulin-dependent diabetes according to criteria for diabetic ketoacidosis^42^. Insulin was occasionally administered to prevent critical weight loss. All animals were housed in groups of 3 to 4 animals at a temperature of 21 ± 1 °C with a 12 h light–dark cycle. At the end of experimental period, animals were killed by cervical dislocation under intraperitoneal ketamine/xylazine anaesthesia. Unless stated otherwise, all reagents were obtained from Sigma-Aldrich Chemie GmbH (Seelze, Germany).

### Immunohistochemistry

Whole-mouse testes were fixed in Bouin’s fluid and then dehydrated in a graded series of ethanol and embedded in paraffin. 5 μm thick sections were processed for staining. Sections were de-paraffinised and rehydrated prior to antigen retrieval.

Localization of CML (diluted 1:100, #CML011, Biologo, Germany), AGE (diluted 1:100, ab23722, Abcam, UK) and RAGE (diluted 1:100, ab3611, Abcam, UK) was performed using a monoclonal or polyclonal antibody, respectively. Specific signal of CML was visualized using a M.O.M. kit (PK-2200, Vector Laboratories, USA) and of RAGE and AGE by treatment with a goat alkaline phosphatase-conjugated anti-rabbit antibody (111-055-003, Jackson ImmunoResearch Inc., USA) followed by fuchsin development (K0625, Agilent Technologies, USA). The sections were counterstained with Mayers’ haematoxylin and mounted under Roti^®^-Histokitt (Cat-no.: 6638.1, Carl Roth GmbH + Co. KG, Germany).

For fluorescence staining 6 µm thick testicular cryosections were briefly fixed in 4% fomalin (pH 7.4) and blocked with 2% BAS in PBS for 1 hour. Then sections were incubated with rabbit anti-PKC antibody (diluted 1:200, ab16898, Abcam, UK) and mouse anti-alpha smooth muscle actin (α-SMA) (diluted 1:500, K046, Linaris, Germany) at 4 °C overnight. Specific signal of PKC and α-SMA was visulized using chicken anti-rabbit Alexia Fluor 488 (diluted 1:200, A21441, Invitrogen, USA) and donkey-anti-mouse Alexa Fluor 555 (1:200, A31570, Invitrogen, USA), respectively, at RT for 1 hour. Sections were then washed with three changes of PBS. Nuclei were stained using Draq5^TM^ (diluted 1:1000, #65-0880-92, Invitrogen, USA). After an intensive wash (3 × 30 minutes) images were taken by a Leica confocal microscope (TCS SP2, Germany) with application suite X software. Positive staining for specific antibodies was confirmed by excluding the primary antibody.

### Protein analysis

Prior to protein analysis, tissues were excised, snap frozen in liquid nitrogen and stored at −80 °C until further processing. For protein analysis tissue samples were homogenised in 1 mL ice-cold phosphate-buffered saline (PBS, buffered to pH 7.4) for 30 seconds and the insoluble fragments removed by centrifugation at 12000 × g for 30 min at 4 °C. Pierce^TM^ BCA protein assay kit (Thermo Fisher Scientific, Carlsbad, CA, USA) was used to measure total protein concentrations according to the manufacturer’s instructions for the microplate procedure. Samples were read at 562 nm on a Labsystems Multiscan RC plate reader (LabX, Midland, ON, Canada). Protein samples were used for further analyses described below.

### Protein kinase C (PKC) assay

Isoforms of the serine threonine protein kinase C (PKC) which require diacylglycerol for stimulation were described in the male reproductive tract. Moreover, PKC activity was reported to be increased in diabetic retina of rodents (Brownlee M, 2001). It was measured using a kit (Cat# ADI-EKS-420A) from Enzo Life Sciences (Farmingdale, NY, U.S.A.) based on a solid phase enzyme-linked immuno-absorbent assay (ELISA) that utilizes a specific synthetic peptide generated from a cAMP respone element binding protein with the amino acid sequence: KRREILFRRPFYR as a substrate for PKC precoated to microplate and a polyclonal rabbit antibody that recognizes the phosphorylated form of the substrate. The assay is designed for the analysis of PKC activity in the solution phase. For the measurement of PKC in protein preparations from testis and epididymis the samples were added to the suitable wells, followed by the annexing of adenosine triphosphate to start the reaction. Then, the phosphospecific substrate antibody was annexed to the wells, which bound to the phosphorylated peptide substrate. The peroxidase-conjugated secondary antibody was then bonded to the phosphospecific antibody. Tetramethylbenzidine (TMB) was used as substrate for the peroxidase. Intensity of color conversion of TMB was proportional to PKC phosphotransferase activity. An acid solution stopped the color development reaction, and the color intensity was measured with microplate reader at 450 nm. Crude protein (10 µg) was determined in each sample to normalize the result to the amount of protein.

### D-Sorbitol colorimetric assay

Sorbitol is a six carbon sugar alcohol produced from glucose by aldose reductase. Due to its poor ability to diffuse across the cell membrane sorbitol is trapped in cells and is one of the causes of damage due to osmotic effects in diabetes. D-Sorbitol content in testis and epididymis samples was measured using the D-Sorbitol Colorimetric Assay Kit (Catalog #K631-100; BioVision Inc., CA, USA). D-Sorbitol was oxidized by nicotinamide-adenine dinucleotide (NAD) to D-fructose in the presence of the enzyme sorbitol dehydrogenase (SDH) with the formation of reduced nicotinamide-adenine dinucleotide (NADH) D-Sorbitol + NAD^+ SDH^ D-Fructose + NADH + H^+^. Under the assay conditions, the equilibrium of the reactions was on the side of NAD and D-sorbitol, respectively. However, they were displaced as the formed NADH was removed in a subsequent reaction in which NADH reduced iodonitrotetrazolium chloride (INT) to a formazan with an absorbance maximum at 560 nm. The assay is useful over the range of 0.1–10 nmol of Sorbitol per sample. Crude protein (10 µg) was measured per sample.

### CML ELISA

Total CML concentration was determined using the ELISA Kit for Carboxymethyl Lysine (CML) (#CEB977Ge, CLOUD-CLONE CORP, TX, USA). Briefly, a monoclonal mouse antibody specific to Carboxymethyl Lysine (CML) was pre-coated onto a microplate. A competitive inhibition reaction was launched between biotin labeled and unlabeled CML in standards or samples. After incubation the unbound conjugate was washed off. Next, avidin conjugated to Horseradish Peroxidase (HRP) was added to each microplate well and incubated. The amount of bound HRP conjugate was reverse proportional to the concentration of CML in the sample. After addition of the substrate solution, the intensity of color developed was reverse proportional to the concentration of CML in sample.

### Western blot analysis

Tissue samples were separated by sodium dodecylsulfate – polyacrylamid gelelectrophoresis (SDS-PAGE) and transferred onto PVDF membranes (Cat# 05317-10EA, Immobilon-FL) following standard protocols. After blocking with Odyssey^®^ Blocking buffer, membranes were incubated overnight at 4 °C with the indicated primary antibodies: anti-RAGE (#ab3611, Abcam), anti-OGT (#24083, Cell Signaling) and anti-phospho histone H2A.X (#2577, Cell Signaling) followed by IRDye 800 CW secondary antibodies (P/N 925–32211, LI-COR). Bound complexes were detected using the LI-COR Odyssey^®^ Fc imaging system (LI-COR, Germany). For quantification, the intensities of the total protein were normalised to REVERT^™^ total protein (LI-COR) signal.

### Multiplex analysis

Analysis of ERK1/2 and Akt was carried out using the MILLIPLEX MAP TGFβ Signaling Pathway Magnetic Bead 6-Plex - Cell Signaling Multiplex Assay kit (#48-614MAG, Merck Millipore, Germany). Results are expressed as median fluorescent intensity (MFI) ± SEM.

### Mass spectrometrometric quantification of MG

Methylglyoxal levels in testis and epididymis were determined by liquid chromatography with tandem mass spectrometric detection (LC-MS/MS), as described previously^43^. Briefly, tissue samples were deproteinized by addition of of 20 μl 10% trichloroacetic acid. 5 pmol of internal standard, [^13^C_2_]-methylglyoxal [Radiopharm. 1990, 28, 1455–1464], was added and the methylglyoxal derivatized by incubation with 100 µmol/l 1,2-diaminobenzene, 100 μmol/l diethylenetriaminepentaacetic acid, and 0.3% (v/v) sodium azide for 4 hrs in the dark. The samples were then separated by reverse phase LC on a Waters® Acquity BEH C18 column (1.7 μM, 2.1 × 50 mm) using an Acquity UPLC class I liquid chromatography system (Waters®) and detected using an XEVO TQ-S tandem quadrupole mass spectrometer (Waters®) in positive electrospray ionization mode. Analyte detection was performed using multiple reaction monitoring (MRM) as described previously^43^.

### qRT-PCR

Total RNA was extracted using the RNeasy Mini Kit from QIAGEN (Hilden, Germany) according to the manufacturer’s instructions. Briefly, testicular and epididymal tissue was excised, snap frozen in liquid nitrogen and stored at −80 °C prior to analysis. For RNA extraction, tissue was thawed, placed in a 2 ml tube holding 1 ml of RLT lysis buffer, and homogenized using a tissue ruptor. Lysed tissue was transferred into a fresh 1.5 ml tube and centrifuged at 13.000 rpm for 10 min. 350 µl supernatant was transferred into a fresh 1.5 ml tube and mixed with 350 µl 70% ethanol. Next, the mixture was transferred onto a RNeasy Mini Spin Column and centrifuged for 1 min. at 13.000 rpm. The flow through was discarded and 500 µl of RW1 buffer added onto the column, before further centrifugation. Again, the flow through was discarded and 500 µl of RPE buffer added onto the column, before further centrifugation. Next the column was washed twice using 500 µl of 80% ethanol, followed by centrifugation. Last, 20 µl RNase free water were added onto the column, incubated for 3 min. and the RNA eluted by centrifugation for 2 min. at 13.000 rpm. RNA yield was quantified using a NanoDrop spectrophotometer (NanoDrop products, DE, USA). Total RNA (1 µg) was reversed transcribed in 20 µl reactions using the Superskript III VILO Kit (Invitrogen, Carlsbad, CA, United States). Real-time PCR amplifications were performed using the IQ SYBR Green Supermix (Bio-Rad Laboratories GmbH, Munich, Germany) on the StepOne Plus real-time PCR system (Applied Biosystems, Waltham, MA, USA). Each well contained 5 µl SYBR Green, 3.2 µl RNase free H_2_O, 0.3 µl Primer, and 1.5 µl cDNA template. Cycling conditions were 95 °C for 10 min, followed by 40 cycles at 95 °C for 15 sec, 60 °C for 30 sec, and 72 °C for 30 sec. After amplification a melting curve analysis was performed to analyze the specificity of the products using the following conditions: 95 °C for 30 sec, 60 °C for 30 sec, followed by 80 cycles at 60 °C for 10 sec. The expression of each of the genes was measured in triplicate for each sample. PCR signal of the target transcript was normalized to the geometric mean of two reference genes (*β-Actin* and *peptidylprolyl isomerase A*) and expression levels were assessed by relative quantification using the 2^−ΔΔCt^ method. Data are expressed as the fold-change in gene expression relative to wildtype control transcripts. See Supplementary Table 1 for primer sequences.

### Statistical analysis

Values in tables and graphs are expressed as mean ± SEM (unless otherwise specified). N-numbers are given in graph bars indicating numbers of animals per group. Data were checked for normality of distribution using the Shapiro-Wilk normality test. Where data showed normal distribution unpaired t-test was performed for comparison between two groups and two-way ANOVA was used to test the effect of diabetes between several groups when multiple time-points were considered. Where the overall ANOVA showed significant differences among means, Tukey’s *post-hoc* comparisons between treatments were performed. When one or more groups failed the normality test, samples were analysed using the Kruskal-Wallis test. Where the Kruskal-Wallis test showed significance, Dunn’s *post-hoc* comparisons between treatment and controls were performed. To test the correlation between two parameters Pearson correlation analysis was performed for normally distributed data (Graph Pad Prism 6 (GraphPad Software, CA, USA)).

## Supplementary information

Supplementary Information
