# Supplementary material for: Hyperglycemia induces spermatogenic disruption via major pathways of diabetes pathogenesis
Source: Sci Rep. 2019 Sep 10;9:13074. doi: 10.1038/s41598-019-49600-4 (PMC6736974; doi:10.1038/s41598-019-49600-4)
Supplement: Supplementary file 1 — Supplementary Information [file 41598_2019_49600_MOESM1_ESM.docx]

**Hyperglycemia induces spermatogenic disruption via major pathways of diabetes pathogenesis**

Constanze C. Maresch^1,2,*, +^, Dina C. Stute^2, +^, Thomas Fleming^3^, Jihong Lin^4^, Hans-Peter Hammes^4^, Thomas Linn^2^

^+^these authors contributed equally to this work

^1^Clinic of Urology, Pediatric Urology, and Andrology, Justus-Liebig-Universitiy, Giessen, Germany; ^2^Clinical Research Unit, Centre of Internal Medicine, Justus-Liebig-University, Giessen, Germany; ^3^German Center for Diabetes Research (DZD), Neuherberg, Germany; ^4^ V. Medical Dept., Medical Faculty Mannheim, University of Heidelberg, Mannheim, Germany

^*^ Correspondence to Constanze C. Maresch, Department of Urology, Pediatric Urology, and Andrology, Justus-Liebig-University, Schubertstrasse 81, Giessen, Germany, E-mail address: Constanze.C.Maresch@chiru.med.uni-giessen.de


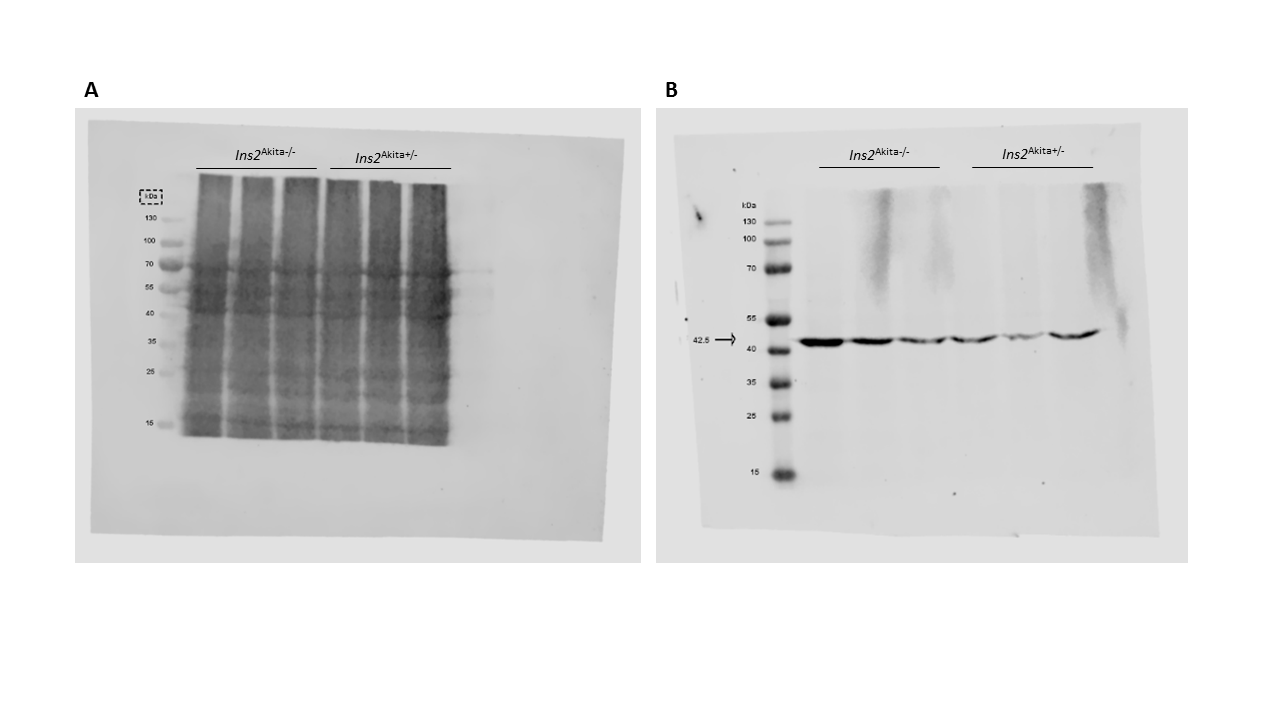


**Supplementary Fig. 1. Original Western Blots used in this study (12 weeks old mice).** Equal amounts of testicular protein lysate of three representative samples of Ins2^Akita-/-^ and Ins2^Akita+/-^ mice were loaded in each lane and separated in a 10% SDS-polyacrylamide gel, transferred to a PVDF membrane, and then imaged with the Odyssey® FC Imaging System in the 700 nm and 800 nm channel. (A) REVERT^TM^ Total Protein Staining. (B) RAGE staining (ab3611, Abcam).


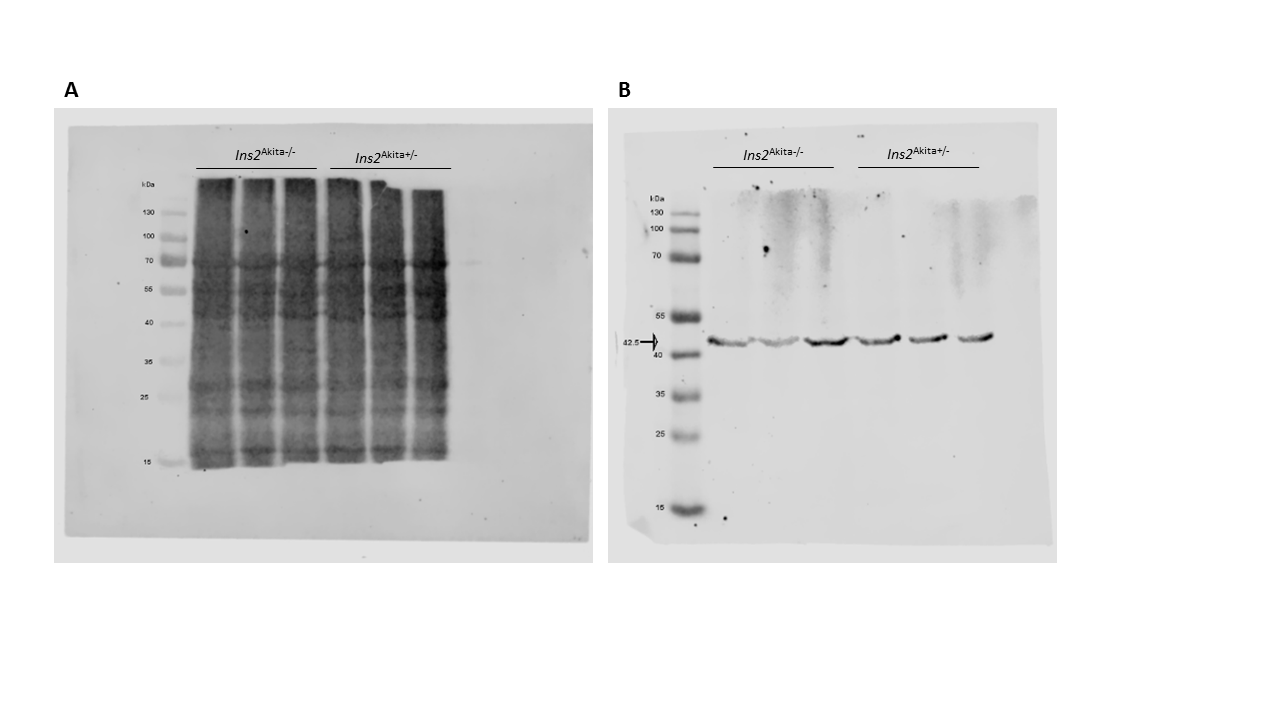


**Supplementary Fig. 2.** **Original Western Blots used in this study (24 weeks old mice).** Equal amounts of testicular protein lysate of three representative samples of Ins2^Akita-/-^ and Ins2^Akita+/-^ mice were loaded in each lane and separated in a 10% SDS-polyacrylamide gel, transferred to a PVDF membrane, and then imaged with the Odyssey® FC Imaging System in the 700 nm and 800 nm channel. (A) REVERT^TM^ Total Protein Staining. (B) RAGE staining (ab3611, Abcam).

**Supplementary Table 1.** Oligonucleotides used for qRT-PCR in this study.

| **PCR primer** | **Forward primer** | **Reverse primer** |
| --- | --- | --- |
| *β-actin* | CACAGCCTGGATGGCTACGT | CGTGAAAAGATGACCCAGATCA |
| *ppiA* | GCAAGCATGTGGTCTTTGGG | TTGATGGGTAAAATGCCCGC |
| *Claudin11* | CTACGTGCAGGCTTGTAGAGC | GGCACATACAGGAAACCAGATG |
| *N-Cadherin* | GGAATCCCGCCTATGAGTGG | CGTCTAGCCGTCTGATTCCC |
| *Gata-4* | CACCCCAATCTCGATAT | GCACAGGTAGTGTCCCGTC |
| *Occludin* | CCAGGTGAGCACCTTGGGAT | TTCAAAAGGCCTCACGGACA |
| *RAGE* | GAAAGCCCTCCTGTCAGCAT | TCTCCGCTTCCTCTGACTGA |
| *ERK1* | CTTCAACCCAAACAAGCGCA | CCATGTCGAAGGTGAATGGC |
| *ERK2* | TCAGTTTGTCCCCTTCCATTG | TCCACTCCCACAATGCACAC |
| *Rela* | ATCATCGAACAGCCGAAGCA | TGATGGTGGGGTGTGTCTTG |
| *Nrf-2* | AAGAATAAAGTCGCCGCCCA | AGATACAAGGTGCTGAGCCG |
| *CDC42* | GCAAAAGGGAATGTGGTCTGG | TCCACCTACGGCACAAACAG |
| *IκBκB* | ACTCCAAAGTCCGGCAGAAG | TCACGGGGTATGTGTGAACG |
| *NFκBIA* | GGGTGATTCGGCTGTTGTCT | CCACTGAACACCTGGCTCTT |
